# Warmer autumns and winters could reduce honey bee overwintering survival with potential risks for pollination services

**DOI:** 10.1038/s41598-024-55327-8

**Published:** 2024-03-25

**Authors:** Kirti Rajagopalan, Gloria DeGrandi-Hoffman, Matthew Pruett, Vincent P. Jones, Vanessa Corby-Harris, Julien Pireaud, Robert Curry, Brandon Hopkins, Tobin D. Northfield

**Affiliations:** 1 Washington State University, Pullman, WA USA; 2 United States Department of Agriculture ARS, Carl Hayden Bee Research Center, Tucson, AZ USA; 3 Tree Fruit Research and Extension Center, Washington State University, Wenatchee, WA USA; 4 Crystal River Consulting, Tucson, AZ USA

**Keywords:** Climate change, Honey bee overwintering, Pollination services, Cold storage, Climate-change impacts, Projection and prediction

## Abstract

Honey bees and other pollinators are critical for food production and nutritional security but face multiple survival challenges. The effect of climate change on honey bee colony losses is only recently being explored. While correlations between higher winter temperatures and greater colony losses have been noted, the impacts of warmer autumn and winter temperatures on colony population dynamics and age structure as an underlying cause of reduced colony survival have not been examined. Focusing on the Pacific Northwest US, our objectives were to (a) quantify the effect of warmer autumns and winters on honey bee foraging activity, the age structure of the overwintering cluster, and spring colony losses, and (b) evaluate indoor cold storage as a management strategy to mitigate the negative impacts of climate change. We perform simulations using the VARROAPOP population dynamics model driven by future climate projections to address these objectives. Results indicate that expanding geographic areas will have warmer autumns and winters extending honey bee flight times. Our simulations support the hypothesis that late-season flight alters the overwintering colony age structure, skews the population towards older bees, and leads to greater risks of colony failure in the spring. Management intervention by moving colonies to cold storage facilities for overwintering has the potential to reduce honey bee colony losses. However, critical gaps remain in how to optimize winter management strategies to improve the survival of overwintering colonies in different locations and conditions. It is imperative that we bridge the gaps to sustain honey bees and the beekeeping industry and ensure food and nutritional security.

## Introduction

Pollinator losses have severe repercussions on ecosystem stability, agricultural production, and food security, in addition to important economic consequences^1,2^. Pollination by bees facilitates the production of ≈ 75% of global crops^3^, especially nutritionally significant fruits, nuts, and vegetables. Worldwide, pollinator populations have steadily declined^4–6^, leading to concerns about global food security and highlighting the need to safeguard pollinators for human and ecosystem well-being^7^. Currently attributed causes of pollinator declines include changing land-use patterns and habitat loss^4,8^, pest and disease pressures^9–11^, pesticide exposures^12^, and lack of forage^13,14^, all exacerbated by climate change^15,16^. Research on climate change effects on pollinators have focused on feral populations and reported species redistribution, asynchronicity in plant-pollinator relationships, and reduced forage availability^17,18^. Less emphasis has been placed on climate change impacts on honey bee population dynamics.

Recent studies linking weather with managed honey bees have shown correlations between monthly meteorological variables and colony losses. Summer temperatures have been associated with winter mortality^19^ and extreme weather exposure has been linked to higher colony losses^20^. Years with warmer and drier conditions especially during the winter were correlated with higher overwinter colony losses^21,22^. Warmer winters also can cause brood rearing to start earlier in colonies. While an earlier start to brood rearing might seem advantageous to colony growth, the amount of brood that can be reared will be constrained by the size of the overwintering population and food availability. Earlier brood rearing might also produce larger populations of parasitic Varroa mites* (Varroa destructor* Anderson and Trueman) that reproduce in brood cells^23^. The mite transmits viruses and shortens the lifespan of adult bees^9,24^. In a field study, higher mite populations resulting from the earlier start in brood rearing negatively affected colony growth throughout the summer and fall.

Based on these recent studies, there are potentially many subtle, yet important effects of warmer autumn and winters associated with climate change^16,25^. However, climate change impact studies on honey bees are limited, and the impacts of warmer autumn and winter temperatures on colony population dynamics and age structure as an underlying cause of reduced colony survival have not been examined. We hypothesize that warmer autumns and winters could affect colony population dynamics and age structure and contribute to colony losses even in the absence of other stressors.

Honey bee colonies are self-organizing^26^, and comprised of a single queen, brood (larvae and pupae), hundreds of drones (males), and thousands of non-reproductive adult female worker bees. The queen can lay more than a thousand eggs per day that develop into adult worker bees in ≈ 21 days. Workers perform various age-based behaviors; the youngest workers are ‘nurse bees’ that care for larvae, and age into ‘house bees’ that build comb, process food, and defend the hive. The older workers forage for nectar, pollen, propolis, and water when the temperatures, precipitation and wind speed are suitable for flight. Foraging activity is negatively correlated with worker longevity^27,28^ because of the physiological stress of flight^29,30^. Colony age structure is seasonal with large amounts of brood and adult workers of various ages occurring in the spring and summer. In the autumn, brood-rearing slows and eventually ends as colonies prepare for winter. In higher latitudes, colonies go into winter with only adults that have transitioned into diutinus or ‘winter bees’ that are adapted to periods of confinement in the hive. Winter bees differ anatomically and physiologically from those in spring and summer. Among the differences, winter bees have greater longevity than spring and summer bees^31,32^. The transition from summer to winter bees in temperate regions occurs in the fall and is associated with the cessation of brood rearing^33^. When temperatures fall below 10 °C, the bees form a thermoregulated cluster in the hive. While in cluster, egg laying and brood rearing can resume in late January^34^, restarting the annual colony cycle.

The size and age structure of the winter cluster are critical to colony survival in the spring^23^. When the bees are in winter cluster, colony sizes remain relatively stable. However, warming daytime temperatures in spring stimulate flight activity causing mortality, especially in the older foraging bees. When forager mortality exceeds the emergence rates of new adults, the worker population declines. The period of colony decline is called ‘spring dwindling’^35^. As spring progresses and brood-rearing increases, the trend ultimately reverses, and the colony grows. Population declines during the spring dwindling period depend on the age structure of the overwintering adult population^36^, and can be particularly severe leading to colony failures if the overwintering population is composed primarily of older workers. One way that the winter cluster can be comprised of older bees is if weather conditions in autumn cause extended periods of flight, thus physiologically aging the bees going into the winter cluster.

To examine the effects that warmer fall and spring temperatures due to climate change might have on overwintering colony dynamics, we simulated honey bee populations under future climate projections using a colony population dynamics model. While climate change impact studies across agricultural and ecological fields ubiquitously utilize gridded future projections of meteorological variables from an ensemble of general circulation models and greenhouse gas concentration pathways^37^, similar applications are not yet prevalent in the context of quantifying impacts on honeybee population dynamics. Our objectives were to (a) quantify the effect of warmer autumns and winters on honey bee foraging activity, the age structure of the overwintering cluster, and spring colony losses, and (b) evaluate management strategies to mitigate the negative impacts of climate change. We assume no nutritional, pest, or heat stressors, isolating the effect of warmer autumns and winter temperatures on colony dynamics.

We focus on the Pacific Northwest of the United States (PNW) as a case study given the diversity of climatic conditions in the region (coastal, temperate, arid and cold conditions as per the Koppen Climate classification^38^), historically successful overwintering with lower colony losses relative to other regions (USDA NASS colony loss statistics summarized by region in Insolia et al.^20^), and prevalence of high-value crops dependent on honey bee pollination (e.g., tree fruit, berries, vegetable crops and alfalfa). To address objective b, we evaluate an emerging management strategy that appears promising for a warming climate—overwintering colonies in temperature-controlled indoor facilities^39,40^. Indoor storage prevents extended foraging by maintaining colonies at a steady temperature of 4 °C during autumn and winter. Recent findings indicate that cold storage can alleviate some of the ambient temperature effects on age structure and increase overwintering survival^39^.

We conducted simulations with the VARROAPOP colony population dynamics model^41^ using an ensemble of climate model projections under two representative concentration pathways (RCPs) with higher (RCP 8.5) and lower (RCP 4.5) greenhouse gas concentrations to address our objectives and determine colony age structure and potential colony losses in a changing climate with warmer autumns and springs. We then evaluated the effectiveness of overwintering in temperature-controlled cold storage facilities as a strategy to improve overwintering survival despite climate change. Our findings are applicable beyond PNW to similar regions with historically good overwintering conditions but that face an increasing likelihood of extended flight weather in the autumn and winter.

## Methods

### Input data

#### Meteorological inputs

Historical simulations (1950–2005) and future climate projections (2007–2099) were based on the Coupled Model Intercomparison Project 5 (CMIP5)^37^ general circulation model results which were bias-corrected and downscaled to the 1/24 degree resolution by the Modified Multivariate Adaptive Constructed Analog (MACA) method^42^, and subsequently re-gridded to the 1/16 degree (∼6 km) resolution by linear interpolation for computational efficiency. Daily maximum and minimum temperature, precipitation, and wind speed variables were used from the spatiotemporally complete 1/16th degree gridded data product. Nineteen climate models under two representative concentration pathways (RCPs) were used for a total of 38 projections. The 19 models used in this study were bcc-csm1-1, bcc-csm1-1-m, BNU-ESM, CanESM2, CCSM4, CNRM-CM5, CSIRO-Mk3-6-0, GFDL-ESM2G, GFDL-ESM2M, HadGEM2-CC365, HadGEM2-ES365, inmcm4, IPSL-CM5A-LR, IPSL-CM5A-MR, IPSL-CM5B-LR, MIROC5, MIROC-ESM, MIROC-ESM-CHEM, and MRI-CGCM3. The RCP 4.5 scenario assumes a stabilization or reduction of greenhouse gas emissions starting around mid-century while the RCP 8.5 is an extreme “no climate policy” scenario that assumes increasing emissions until the end of the century and is associated with relatively higher temperature increases post-mid-century compared with RCP 4.5^43^.

#### Other data

In order to place the mapped results in the context of lower elevation cropland areas, the 1 arcsecond (~ 30 m) resolution LANDFIRE elevation dataset^44^ was aggregated to the 1/16 degree (~ 6 km) resolution of the meteorological input data. This was mapped for visual context and no analysis was performed utilizing this.

### Calculation of overwintering flight hours

The number of honey bee flight hours on a given day is calculated from the meteorological data utilizing the process used in the honey bee population dynamics model used in this study^41^. It is the number of hours in a day between sunrise and sunset with temperatures between 12 and 43.33 °C while also having low precipitation (< 5 mm) and low wind speed (< 8.94 m/s). Winter flight hours are defined as all flight hours occurring between November 1st and January 31st of the following year.

### Honey bee population dynamics model

The honey bee colony dynamics were simulated by the VARROAPOP model^29^, which is a mathematical model of varroa mite and honey bee population dynamics. VARROAPOP was originally developed and validated in 2004, and has been applied in multiple simulation contexts^45–47^ and found to generate predictions consistent with empirical data from field studies^48–50^. While the model can simulate both the varroa mite and honey bee dynamics, in our application, we do not include Varroa or any other factors in the simulations that might limit colony growth (e.g., poor queen egg laying or reduced worker bee longevity due to nutritional stress, disease, or pesticides). Consequently, the colony dynamics we simulate are products of weather conditions and conservative as other stress factors will likely further reduce colony sizes and chances of survival.

Details regarding the model and assumptions can be found in the original publication^41^ and in the GitHub code repository. Briefly, model predictions incorporate weather conditions and use daily maximum and minimum temperatures, precipitation, and wind speed to generate daily estimates of adult bee (colony size) and brood populations (egg, larvae and pupae). Based on days since emergence from the pupal stage, adult workers are divided into non-foraging ‘young bees’ (adults < 21 days old) and foragers (workers > 21 days old). Colony growth is driven by the queen’s daily egg-laying rate which is determined by her age, maximum egg-laying rate, colony size, and weather conditions, specifically temperature and photoperiod. The number of eggs laid each day are aged as cohorts to adult bees. For worker bees, the time spent in each life stage is three days for an egg, six days for a larva and 12 days as a pupa. On day-22 after the cohort of eggs is produced, it is added to the adult worker population in the colony. The cohort remains in the non-forager ‘young bee’ population for 21 days and then becomes a forager. In spring and summer, the model simulates colony growth due to greater daylight hours and warm temperatures. In the fall, the decreasing temperatures and daylight hours cause a reduction in brood rearing until the model predicts that brood rearing ends in temperate areas by October or November. When conditions are not favorable for flight, adult bee aging stops until foraging resumes. Low temperatures can occur for long periods during late fall and winter thus increasing the lifespan of adult bees in the winter. The combination of an end of brood rearing and increased adult bee longevity simulates the development of winter bees in the colony.

In addition to the queen’s egg laying rate, model predictions of colony growth are affected by the longevity of adult workers. In the original VARROAPOP model, worker longevity was determined by the number of days a cohort of adult workers could forage before being removed from the colony population (forager lifespan). Forager lifespan was specified at the start of the simulation and depended on the occurrence of weather conditions that were conducive for flight. A modification in the current version of VARROAPOP is that foragers are aged more conservatively. In previous versions of the model, a day having weather conditions conducive for flight was considered a full foraging day regardless of the number of hours when the weather was favorable for flight. The assumption was modified in the current version of VARROAPOP to score a full foraging day as occurring only on the summer solstice under ideal flight conditions (no reduction in flight due to temperature, wind speed, cloud cover (i.e., solar radiation) or rainfall). As conditions each day depart from the hours of ideal flight conditions, foragers are aged as having partial foraging days. When the total of the partial foraging days equals the assigned forager lifespan, the cohort is removed from the colony population. Therefore, in the fall or early spring when flight weather might occur for only a few hours each day, it will take more calendar days than the assigned forager lifespan to cause a cohort to be removed from the population. This nuance was important to include in a climate change context to prevent overestimating the impacts of foraging weather in late fall and early spring.

The current version of VARROAPOP also includes an option for placing hives in cold storage for intervals during the simulations. On the chosen cold storage start date, foraging stops as does the aging of adult workers so that the colony age structure is stable during the cold storage period. If there are cohorts of larvae in the colony when cold storage begins, the larvae are removed from the population, while pupae are aged and emerge as adults. Egg-laying resumes in late January as in outdoor colonies consistent with observation under cold storage experiments^51^. When the colony is removed from cold storage and weather conditions are conducive for flight, adult worker aging resumes with foragers from the previous fall to be the first to be removed from the colony population.

### Simulation framework

The model described in section “Honey bee population dynamics model” is driven by the daily historical and future meteorological inputs described in section “Input data”. The simulations are run independently for each 1/16th degree grid noted in section “Input data”. Each year is considered an independent weather realization because we are interested in isolating the impacts of warmer autumns and winters on spring losses. Therefore, each year, we reset the colony age structure on June 15th to conditions a beekeeper who manages a colony would ensure. Specifically, we estimated the median age structure on June 15th in Omak, Washington (which has good overwintering conditions) and applied it to our study domain. We also replaced the queen each year to optimize egg-laying and set the queen strength to a maximum potential egg-laying capacity of 1500 eggs per day. The model is run under conditions of no nutrient limitations and the absence of pests and pathogens.

### Aggregation process

Results are presented either as continuous time-series with the median and range across models or as aggregations of these time series across decadal time periods, as appropriate. The decadal time periods considered are: Historical (baseline) (1975–2005), near-future (2025–2055), mid-future (2045–2075), and distant-future (2065–2095). When data are summarized across models and timeframes, they are presented as medians over the ensemble of models and the years in the timeframe. In addition, more detailed analysis is shown in some sections for two locations showing contrasting behavior. Richland, WA (46.28° N, 119.28° W) with warmer winters at a lower elevation of 117 m and Omak, WA (48.41° N, 119.53° W) with cooler winters at a higher elevation of 257 m. Both locations grow tree fruit requiring honey bee pollination services.

## Results

### Autumn and winter flight hours

**Figure 1:**
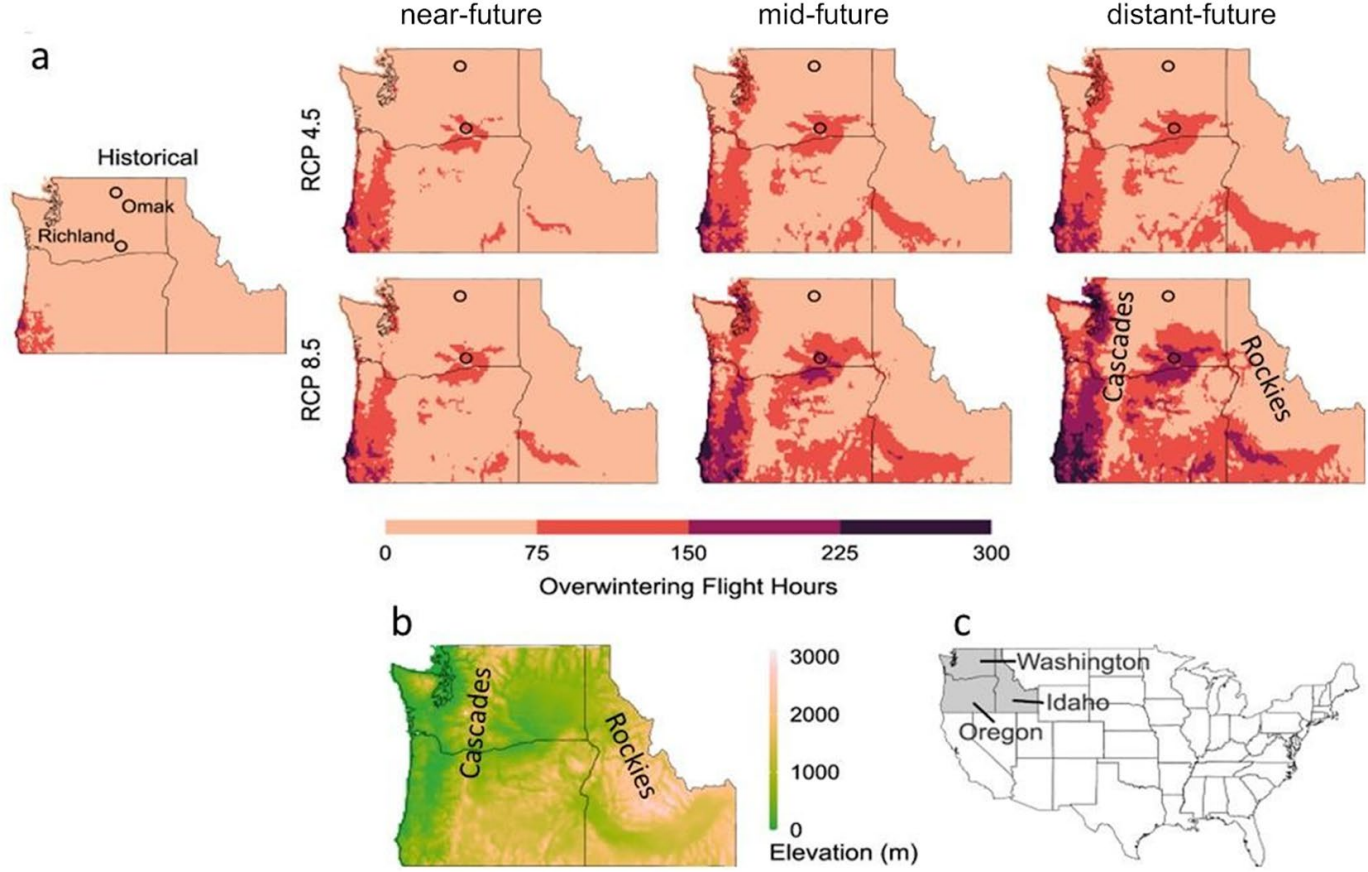
(**a**) The median annual flight hours between November 1st and January 31st for Historical, and near-future, mid-future, and distant-future time frames for two RCPs, (**b**) elevations with Cascade and Rocky Mountain ranges marked, (**c**) Map of the continental United States with highlighted study area (grey). The historical panel of ‘a’ marks two locations in Washington State, Omak, and Richland, which are referenced in the text. Note that the elevation data is not used in any analysis and is provided solely for visual context.

Historically, most parts of the PNW have limited periods in autumn and winter with temperatures that are conducive for honey bee flight (Fig. 1a). However, temperatures favorable for flight increase in frequency and spatial extent under both RCPs. Areas that retain low autumn temperatures that limit flight correspond to northern latitudes and higher elevations (Fig. 1b) that are snow-covered for much of the winter, including the Cascades and Rocky Mountain ranges.

### Effect of autumn and winter flight on spring honey bee colony size

**Figure 2:**
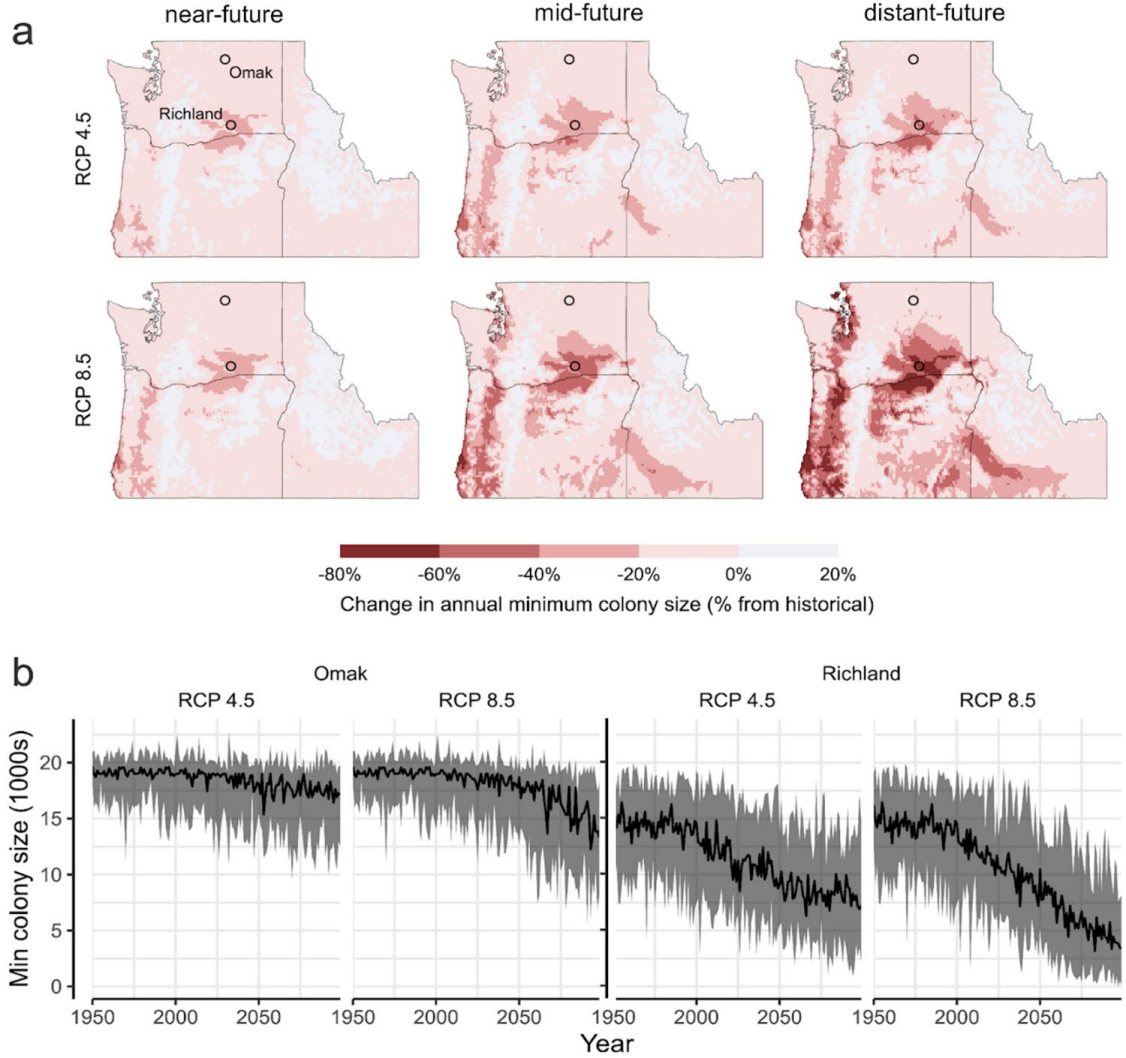
(**a**) Percentage change in annual minimum colony size in the future time frames as compared to the historical time frames. Medians across 19 general circulation models are shown for each RCP. The years averaged for each time frame include 1975–2005 (historical), 2025- 2055 (near-future), 2045–2075 (mid-future), and 2065–2095 (distant-future). (**b**) The time series of annual minimum colony size for two representative locations, Omak and Richland, and two RCPs. The black line in the middle is the median across all 19 general circulation models and the shaded area is reflective of the range of values from models.

Increased autumn and winter flight lead to smaller colonies the following spring (Fig. 2a) based on model simulations. Annual minimum colony size (defined as the lowest the colony size drops to yearly) by mid-century is less than half of historical values in many areas. Some northern and high elevation areas—away from agricultural lands—continue to have good overwintering potential with minimal to no reductions and even slight increases in the annual minimum colony size. Contrasting changes in annual minimum colony sizes are exemplified in two locations; Omak and Richland (Fig. 2b). For Omak, in northern Washington, the adult population at the lowest point of spring dwindling reduces 2% (near-future), 6% (mid-future), and 8% (distant-future) for RCP 4.5 and 4% (near-future), 10% (mid-future), and 20% (distant-future) for RCP 8.5 as compared to historical conditions. In contrast, in Richland in southern Washington, reductions for RCP 4.5 are 26% (near-future), 38% (mid-future), 42% (distant-future) and 32% (near-future), 52% (mid-future), 67% (distant-future) for RCP 8.5, and reach levels < 9000 adult bees/colony by mid-century for both RCP 4.5 and 8.5, and < 5000 adult bees by end-of-century under RCP 8.5 (Fig. 2b). Large decreases in minimum colony size point to an increased risk of colony failure^52^.

### Effects of autumn and winter flight on colony age structure

**Figure 3:**
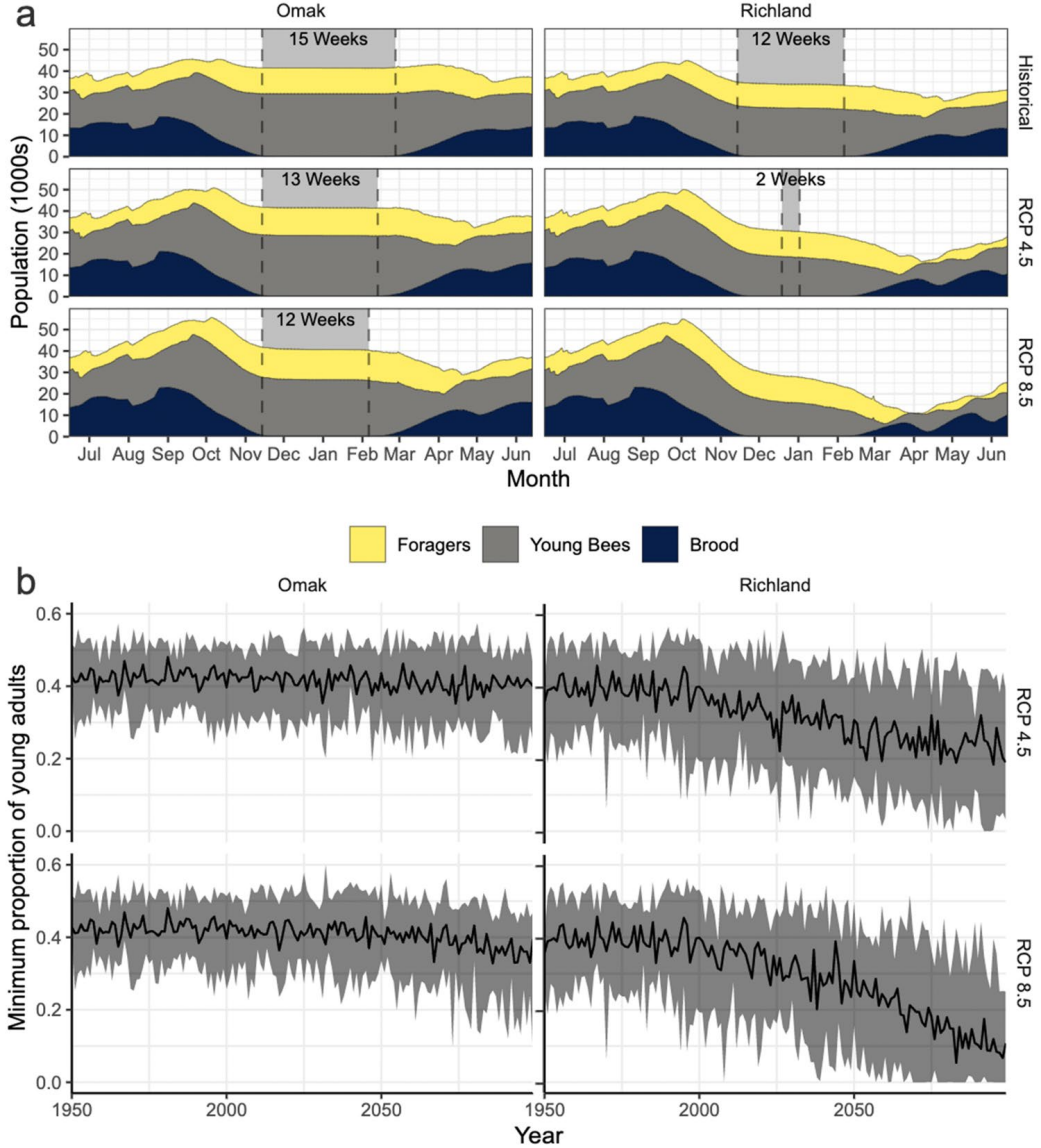
(**a**) Stacked populations of brood and adults (split as young bees with no flight experience and older foragers with flight experience). The simulation starts in July and ends in June of the following year. The median colony population across all the years in the time frames (1980–2006 for historical and 2065–2095 for distant-future) and the 19 general circulation models are displayed for two RCPs and two representative locations. The dotted vertical lines and gray shading is indicative of the weeks when colony population and age structure (relative numbers of bees in different ages) are stable with minimal changes in the adult population (change less than 300 per week). (**b**) Time series plots of the minimum proportion of young adults/total adults, by year. The time series ranges from 1950 to 2099. The black line in the middle is the median across all 19 general circulation models and the shaded area is reflective of the range of projected values. Two RCPs are displayed for the representative Omak and Richland locations.

Three aspects of climate change impacts on colony dynamics are apparent when comparing simulations with future and historical conditions (Fig. 3a). These are best illustrated using the distant-future simulations in Omak and Richland, as contrasting examples showing the largest changes. First, there is an increase in the rate of autumn colony population decline particularly in Richland (October/November in Fig. 3a). This is due to extended future autumn flight-conducive weather and related mortality. Second, the length of time with a stable winter population decreases (vertical gray shaded area). Third, spring temperatures conducive for flight commence before brood-rearing begins (compare the time of the end of the vertical gray shaded area of stable populations with the dark blackish blue shaded area for when brood-rearing resumes). The relative differences in the magnitude and timing of these changes result in contrasting minimum colony size outcomes.

Historically, overwintering colony size and age structure were stable in Omak and Richland, with bees confined to the hive for 12–15 weeks. By the time flight-conducive weather resumed in the spring, brood-rearing was underway and the new generation of adult bees could compensate for forager mortality. Under future climates, the length of time with stable winter populations decreases in both locations, but is more pronounced in Richland, from 12 weeks historically to 2 weeks by end-of-the-century under RCP 4.5 and a complete loss under RCP 8.5. In Omak, there is a minimal lag between the period of stable winter population and when brood-rearing is initiated, even in future conditions; thus spring dwindling is less impacted. The spring decline in the adult population in Richland occurs because temperatures are conducive for flight before newly emerged adult bees are available to replace dying foragers. This dynamic initiates a corresponding decrease in the brood (dark blackish blue shading in Fig. 3a) in early May in Omak and April in Richland. In colonies, adult bees are required to rear brood and this is simulated in the model by making egg-laying rates and brood production functions of the adult population size.

Foraging in late autumn and winter shifts the colony age structure toward older bees, so that in the following spring non-foraging (young) bees comprise a lower proportion of the adult population. In Omak, the minimum proportion of young to total adults remains relatively stable (RCP 4.5) or declines slightly from historical levels by the end of the century (RCP 8.5) (Fig. 3b). This modest change indicates that future colony population increases after spring dwindling will resemble historical conditions where there is sufficient emerging brood to replace dying foragers, causing the population to rebound and grow by May (Fig. 3a). In contrast, Richland has a decreasing proportion of young adult bees with no flight experience and physiological stress coming out of the winter; the minimum proportion of young adults to total adults progressively decreases from about 0.4 historically to about 0.2 by mid-century and less than 0.1 by the end-of-century for RCP 8.5 and to about 0.2 for RCP 4.5. Therefore, in places like Richland, colonies have fewer young bees to mitigate spring dwindling and support the robust brood-rearing needed to avoid colony failure^36^.

### Cold storage as an overwintering management alternative

While we simulated the effects of overwintering hives in indoor temperature-controlled cold storage for all time frames, here we are illustrating its effectiveness in the worst-case scenario without cold storage—distant-future in Richland. We simulated colonies placed in cold storage on October 15 and removed either on (a) April 1, when pollen from early-blooming plant species is available in the PNW, or (b) January 31st, in preparation for almond pollination. Each year, more than a million hives are moved to California in early February to pollinate ≈ 4000 km^2^ of almonds^53^.

**Figure 4:**
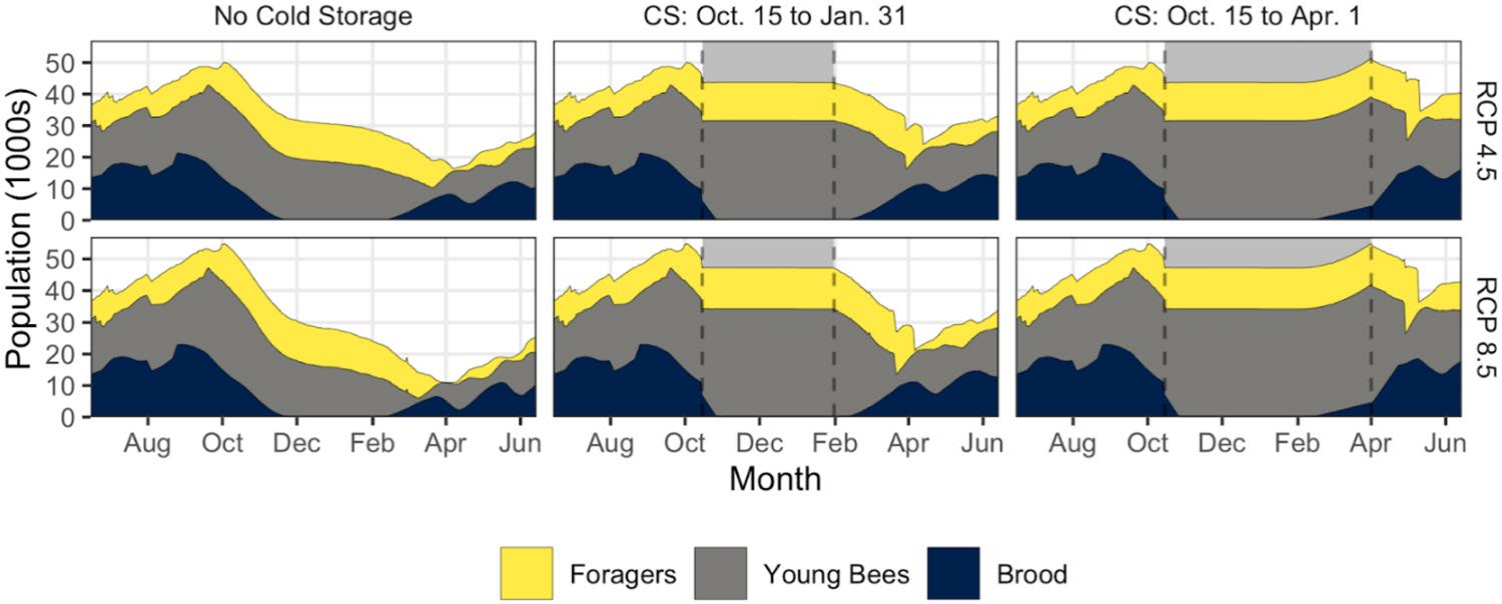
Comparison of the seasonal age structure. Details are similar to Fig. 3 except that this compares three scenarios: no cold storage, cold storage between October 15 and January 31, and cold storage between October 15 and April 1 for the extreme scenario of Richland in the distant-future for two RCPs. Gray regions represent cold storage periods.

Foraging stops while the colony is in cold storage causing adult population size and age structure to remain stable during this period (Fig. 4). For cold storage simulations using RCP 4.5 for the distant-future, the median annual minimum adult populations are 11,269 (ending January 31) and 17,306 (ending April 1) as compared to 8134 with outside overwintering, while the corresponding values for RCP 8.5 are 8963, 17,969, and 4655. Colonies in cold storage until April 1 have populations that are larger than baseline historical values (15,198) during spring dwindling, even in the distant-future. Simulations keeping the hives in cold storage until April 1 generate larger populations than those with hive removal on January 31 because brood-rearing begins in late January while colonies are in cold storage, and new adults emerge prior to the resumption of foraging and simultaneous loss of adult foragers. When cold storage is extended to April 1, the lowest population occurs in mid-May compared to mid-April when overwintered outdoors. The greater number of adults facilitates more brood rearing, and the post-spring-dwindling population increases to higher levels by June compared to colonies overwintered outdoors. Thus, indoor cold storage is a promising management strategy even in a worst-case scenario like distant-future Richland for RCP 8.5, especially when the hives are retained in cold storage for a longer period.

## Discussion and conclusion

Our results highlight that understanding and managing the effects of warmer autumns and winters on honey bee colonies should be an area of emphasis to reduce colony losses. Warmer autumns and winters—projected to occur with higher frequency and in expanding areas in northern latitudes like the PNW—can alter the age structure of the overwintering cluster and limit spring colony growth, thus negatively impacting colony survival even in the absence of other stressors. While historically, most areas in the PNW were suitable for outdoor overwintering, climate change could cause significant geographic expansion of areas that would become unsuitable for successful outdoor overwintering. Altitude and latitude play a role in moderating the level of impact, given that these factors impact temperatures. High elevations and northern latitudes are places where climate change has relatively lower/minimal impacts on colony losses, and might be places for outdoor overwintering. However, these locations, likely forested, may pose logistical challenges to migrating beekeepers due to issues such as accessibility and snow cover early in the season when colonies may need to be moved.

Our simulations show that when colonies forage later in the fall there are greater population declines in the spring. The late fall flights cause a greater proportion of older foragers in the winter cluster that can accelerate adult population decline in the spring. Recovery can be difficult especially if foraging resumes before new adults emerge to replace dying foragers. In our simulations of conditions in Richland under RCP 8.5, there was a period in early spring when all foragers from the previous fall had been removed from the population due to extended periods with conditions that were suitable for flight. When foragers are removed from colonies, younger bees will begin foraging earlier (i.e., precocious foraging). Precocious foragers have reduced longevity further limiting colony growth^54^. As populations decline, brood-rearing decreases and the colony manifests a fundamental attribute of social insect dynamics (i.e., Allee effects) where large colonies rear increasing amounts of brood and get larger, while small colonies rear progressively less brood and eventually fail^55^. Smaller colonies are also less efficient at pollinating early-season crops (e.g., almonds) as foraging activity is related to colony size^56^. Changes in beekeeping practices that mitigating the effects of warmer autumns and winters will become critical by mid-century even in the absence of other stress factors, because the potential for managed colonies to adapt to conditions brought on by climate change is limited for several reasons. Commercial beekeepers source queens from select geographic areas^57^ not representative of the places they will be installed in, and move colonies throughout the country. Moreover, as a non-native species to the US, only a tiny fraction of honey bee diversity is present in the US. To reduce colony losses, adopting alternate management strategies such as overwintering colonies in cold storage or identifying new locations suitable for outdoor overwintering will become essential.

Our simulations assumed no stress from malnutrition, pathogens, parasites, or pesticides, all of which can shorten the life of adult bees. Additionally, simulations used queens with egg-laying potentials that assured colony growth and included annual queen replacement. In this way, our results can be considered ‘best case scenarios’ for the effects of extended periods of autumn and winter flight weather. In the simulations, colony sizes in Richland dropped to a median < 9000 adults during spring dwindling by mid-century under both RCPs and < 5000 by end-of-century for RCP 8.5. Colonies with fewer than ≈5000–9000 adults have little chance of survival^58^, and many areas of the PNW will reach this threshold. Even in simulations where spring colony sizes are above these thresholds, the effects of warmer autumn temperatures put colonies in a precarious position where a slight decrease in queen egg-laying potential or the presence of a pathogen could push the population beyond the point of recovery^36,59^.

Honey bees pollinate many agricultural crops, but perhaps none is more reliant on honey bees than California almonds, which is also the primary source of revenue for the beekeeping industry^60^. Since almonds currently bloom in February, colonies that have a break in brood rearing between autumn and late January will have foraging populations composed primarily of bees that emerged the previous autumn. As climate change shifts the overwintering population age structure towards older bees, colonies could decline rapidly from robust foraging on almond blossoms. The impact of smaller colonies coming out of almonds could extend to the pollination of later-blooming crops and the economic viability of the beekeeping industry. Our simulations do not account for potentially earlier bloom time due to climate change, but if they occurred, there would be less brood present, and even greater colony decline and losses during almond pollination.

Our simulations suggest that overwintering hives in cold storage could be effective in managing the age structure of overwintered colonies and mitigating the effects of warm autumn and winter temperatures. Increasing numbers of beekeepers are using this overwintering management technique^39,40^, however, there is much to learn about optimizing the process. For example, colonies must have bees that are physiologically prepared for wintering (i.e., winter bees), but the environmental factors that cause bees to change physiologically from brood production and growth to the overwintering phenotype are not well understood^33,61,62^. Optimizing the nutrition of winter bees so they can survive the winter and vigorously rear brood before flowering plants become available is important for colony growth in the spring and needs further study. How latitude and environmental conditions trigger the overwintering phenotype and effects from climate change on the transition from summer to winter bees also need additional study to determine the feasibility and optimal timing for placing hives in cold storage.

While advantages exist, cold storage requires energy and has an associated carbon footprint that needs to be considered. However, in the PNW a significant portion of the energy production comes from relatively cleaner hydropower and cold storage structures typically have solar panels on the roof. The land footprint of these structures also tend to be relatively small with the potential for 10,000–80,000 colonies housed in a single structure. Moreover, given longer times in cold storage are more beneficial, any advancement in bloom time under climate change may reduce the benefits of cold storage by forcing bees to be removed out of cold storage sooner than ideal. However, overwintering in cold storage will be significantly better than not even in these conditions.

Predictions from our study are based on managed honeybee colonies that experienced warmer falls and winters in the PNW. However, some of our findings might presage how feral and managed honey bee colonies in other geographic areas will respond to higher fall and winter temperatures. Feral honey bees might adapt by extending or increasing brood rearing in the fall, and restarting brood-rearing earlier in the spring as honey bees do in southern latitudes. However, the potential for such adaptations in temperate areas with periodic freezes is unclear, as flowering plants would need to be available to support brood rearing. Extending the period of brood rearing in the fall could negatively affect the colony, as it might preclude the formation of winter bees and the storage of nutrients in their fat bodies to sustain them during periods of confinement in the hive when the weather turns cold. Feral honey bees also could respond to the changing climate as non-*Apis* bees (e.g., bumblebees) have by local range contraction, and dispersal into previously marginal areas^63,64^. Feral honey bees might disperse to northern latitudes or higher elevations with conditions similar to Omak, as these areas could remain favorable for overwintering. Honey bees also could migrate to southern latitudes and forego overwintering. While feral colonies will need to adapt to survive, managed colonies will need beekeeping practices to adapt to future climate conditions. Methods developed for outdoor overwintering based on conditions where bees are mostly confined in the hive from mid-fall to early spring may not be applicable in regions where future weather will be similar to or warmer than Richland. Instead, overwintering management of colonies in lower elevations and southern regions of the temperate zones will need to include preparations for movement either to more northern or southern latitudes or into cold storage.

While autumn has historically been a time of preparation for winter hibernation and confinement, climate change is making it a crucible that defines the ranges and diversity of species across habitats. For honey bees, climate change will also dictate how colonies are managed in winter to avoid high losses. The success of the management schemes will have broad implications for the socio-economic viability of the beekeeping industry, the production of crops that rely on honey bee pollination, and food and nutritional security.

## Data Availability

The climate data inputs used in this study are available at https://www.climatologylab.org/maca.html. The code for the VARROAPOP model implementation used in this study are available at https://github.com/jpireaud/Beepop/releases/tag/ClimateChangePaperVersion
